# Peripapillary Capillary Network in Methanol Induced Optic Neuropathy

**DOI:** 10.18502/jovr.v17i1.10180

**Published:** 2022-01-21

**Authors:** Kiana Hassanpour, Negin Mohammadi, Hamideh Sabbaghi, Alireza Amirabadi, Mohammad Pakravan

**Affiliations:** ^1^Ophthalmic Research Center, Research Institute for Ophthalmology and Vision Science, Shahid Beheshti University of Medical Sciences, Tehran, Iran; ^2^Department of Ophthalmology, Imam Hossein Hospital, Shahid Beheshti University of Medical Sciences, Tehran, Iran; ^3^Ophthalmic Epidemiology Research Center, Research Institute for Ophthalmology and Vision Science, Shahid Beheshti University of Medical Sciences, Tehran, Iran; ^4^Department of Optometry, School of Rehabilitation, Shahid Beheshti University of Medical Sciences, Tehran, Iran

**Keywords:** Methanol-induced Toxic Optic Neuropathy, Optical Coherence Tomography Angiography, Radial Peripapillary Capillary Network

## Abstract

**Purpose:**

To present the optical coherence tomography angiography (OCT-A) findings of the radial peripapillary capillary (RPC) network in an individual with severe bilateral methanol-induced toxic optic neuropathy (MTON) in comparison to a normal subject and a patient with retinitis pigmentosa.

**Case Report:**

A 35-year-old man with severe bilateral MTON was referred to the neuro-ophthalmology clinic at the Labbafinejad Medical Center. The Angio Vue OCT 3D set of 4.5 × 4.5 mm was used to measure the disc and peripapillary vessel density. Two subjects were examined with the same protocol as controls to determine the effect on the RPC vessel density in multiple scenarios. One of the controls was a healthy individual with the prerequisite matches of age and sex while the second one was a known retinitis pigmentosa (RP) patient. RPC density was measured as 37.7 in the patient with MTON, 46.9 in the RP patient, and 54.7 in the healthy control.

**Conclusion:**

The reduction in the RPC vessel density in a patient with MTON compared to that of a healthy individual and also a patient with RP may be due to the loss of capillaries secondary to the loss of nerve fibers and ganglion cells. Moreover, MTON can be considered an optic neuropathy with direct mitochondrial damage to the endothelial cells of the capillaries.

##  INTRODUCTION

Methanol poisoning could be caused by drinking homemade alcoholic beverages.^[1]^ Patients who survive this life-threatening condition may also suffer other morbidities including methanol-induced toxic optic neuropathy (MTON).^[2]^ Formic Acid, a metabolite of methanol can result in acute retinal ganglion cells injury and edema of the optic nerve. The presence of the intraretinal fluid revealed in the optical coherence tomography (OCT) reports could present an argument for the role of vessel injury as one of the plausible culprits in the pathophysiology of the disease, MTON. Nurieva et al showed a progressive and chronic loss of those axons that survived after methanol poisoning which supports the aforementioned hypothesis.^[3]^ Due to the rarity of these cases, studies are scarce and the exact mechanism behind the progressive axonal loss remains unknown.

To examine retinal vascularity in MTON, fluorescein angiography (FA) is not always feasible because of the concurrent poor general status and high prevalence of renal insufficiency in these patients. Optical coherence tomography angiography (OCT-A) as a noninvasive novel technique for visualization of vascular flow is viable and provides high-resolution images of both retinal and radial peripapillary capillaries.^[4]^ In this report, we present the OCT-A findings of the radial peripapillary capillary (RPC) network in an individual with severe bilateral MTON.

##  CASE REPORT

A 35-year-old man with the chief complaint of bilateral decreased visual acuity following the ingestion of a homemade alcoholic beverage two weeks prior was referred to us. He had been in a coma for two days and had undergone hemodialysis twice during the acute phase. He had previously received the protocol suggested by the author (MP) that included erythropoietin and intravenous steroid.^[2,6]^ Visual loss was detected after the improvement of consciousness. When we first saw him, visual acuity was counting fingers at 30 cm in the right eye and no light perception (NLP) in the left eye. Trace afferent pupillary defect was detected in the left eye. The anterior segment slit-lamp biomicroscopy and Goldmann applanation tonometry results were normal. In the funduscopy, the optic nerves were mildly swollen. Macula and retinal periphery tests were normal bilaterally. The perimetry test was not possible to execute.

**Table 1:** Peripapillary OCT-A parameter in MTON, RP, and healthy control


**Variables**	**MTON vessel density (%)**		**RP vessel density (%)**		**Healthy control vessel density (%)**	
	**Capillaries**	**All**	**Capillaries**	**All**	**Capillaries**	**All**
Whole image	OD	38.4	45.5	45.7	49.5	50.1	56.5
	OS	37.1	43.6	44.7	48	49.9	56.6
Inside disc	OD	39.4	51	39.7	50	45.2	54.7
	OS	34.6	44.5	44.4	53.2	47.0	57.8
Peripapillary	OD	37.7	44.9	46.9	50.2	54.7	60.6
	OS	36	43.3	42.3	45.1	54.1	60.5
Superior hemifield	OD	36.5	44.2	46.9	50.2	54.8	60.9
	OS	39.1	46	39.7	41.9	53.5	61.2
Inferior hemifield	OD	39	45.5	47	50.1	54.5	60.2
	OS	32.7	41.3	45.1	48.5	54.8	59.7
Nasal	OD	34	47	52	
	OS	32	47	63	
Temporal	OD	51	52	54	
	OS	27	41	47	
MTON, methanol-induced toxic optic neuropathy; RP, retinitis pigmentosa							

**Figure 1:**
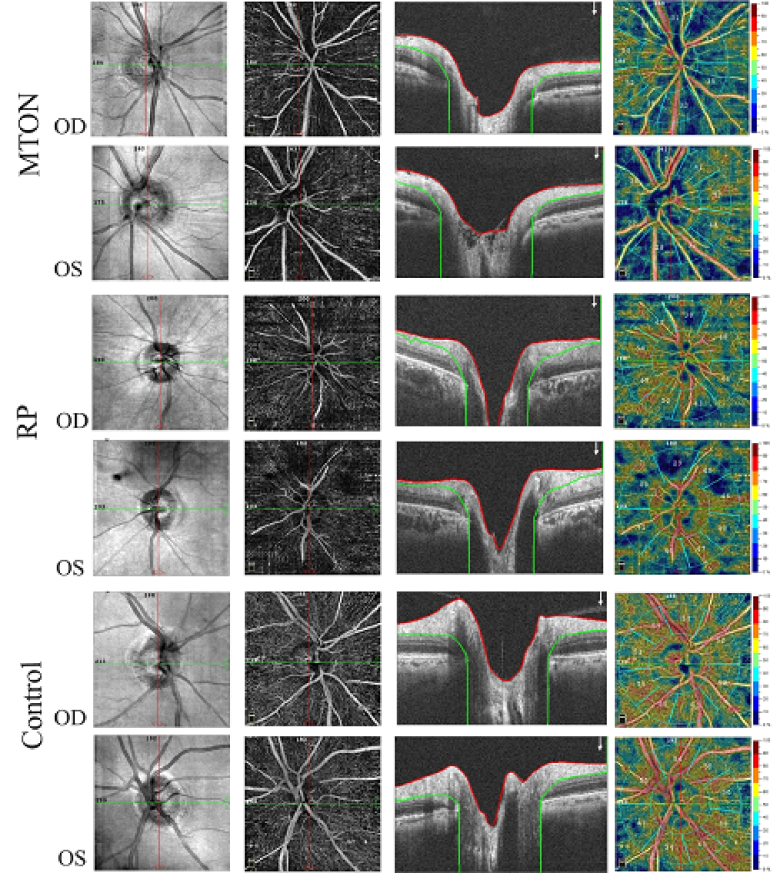
Peripapillary OCT-A images in MTON compared to RP and a healthy individual. From left to right, SLO images, En-face OCT-A (ILM-NFL), corresponding B-scan, vessel density map in MTON, RP patient, and a healthy control. Vessel density analysis has been done between two slabs shown in B-scans.

The OCT-A was performed using XR Avanti Angio Vue OCTA (Optovue Inc., Fermont, CA, USA). The Angio Vue OCT 3D set of 4.5 × 4.5 mm was used to measure disc and peripapillary vessel density. For vessel analysis, a slab between the outer limit of the retinal nerve fiber layer (RNFL) and the internal limiting membrane was made. Two regions of interest (ROI) were defined for measuring vessel density within the area occupied by the vessels. Two elliptical contour lines were used first for defining the disc area which was determined manually and second for corresponding to a peripapillary area with a width of 0.75 mm from the first elliptical line. Two subjects were examined with the same protocol as controls. One of the controls was a healthy individual with the matching prerequisites of age and sex while the second one was a known patient with a history of retinitis pigmentosa (RP), which started from early adulthood and visual acuity of counting fingers at 4 m in both eyes. In examination of the RP patient, it was revealed that he had optic pallor in both eyes as well as arterial narrowing, diffuse retinal degeneration, and peripheral bone spicules.

Capillary peripapillary vessel density was 37.7% in the MTON patient, 46.9% in the RP patient, and 54.7% in the healthy control. Figure 1 shows the OCT-A images of all three cases. In the MTON case, measurements of the OCT-A Angio Vue vessel analysis were all lower, as compared to that of the controls [Table 1]. Peripapillary OCT in the patient with MTON revealed an average RNFL thickness of 148 microns in the right eye and 140 microns in the left eye.

##  DISCUSSION

In this case report, we described the findings of the peripapillary OCT-A in a case of MTON. We discovered the reduction in the RPC vessel density two weeks after the MTON situation was compared to a healthy individual and also to a patient with optic pallor secondary to RP. The possible mechanism explaining the reduced vascular density may be the loss of capillaries secondary to the loss of nerve fibers and ganglion cells.^[5]^ Loss of RNFL and the ganglion cell layer (GCL) happens as a result of two separate mechanisms. Formic acid which is a toxic metabolite produced after methanol ingestion directly enters the ganglion cells and causes severe structural and functional damage. Ganglion cell damage then results in nerve fiber loss. Moreover, the edema subsequent to RNFL damage may cause a compartment syndrome.^[6]^

Various studies investigating OCT-A in different optic neuropathies reported a reduction of peripapillary vessel density, for example, in non-arteritic anterior ischemic optic neuropathy (NAION),^[7]^ optic neuritis,^[8]^ optic atrophy secondary to retinal dystrophies,^[9]^ thyroid eye disease,^[10]^ vitamin deficiency,^[11]^ or Leber hereditary optic neuropathy (LHON).^[12]^ Two possible mechanisms may explain peripapillary vessel density reduction in different optic neuropathies. First, any diseases causing axonal loss leads to reduced metabolic need in the RNFL layer and consequently reduces capillaries through autoregulatory mechanisms.^[5]^ The next mechanism is the direct injury of the capillaries by the acquired disease. While the former is thought to be more prominent in vessel dropouts, the latter can also be highlighted in optic neuropathies with mitochondrial damages such as with LHON which can also have a direct adverse impact on vascular endothelial and vascular smooth muscle cells viability.^[13]^

Examining both eyes of the patient, the eye with more severe visual loss showed lower vessel density in all four quadrants. RNFL thickness cannot be a reliable measure in the acute phase, however, the thickness was slightly higher in the right eye (148 and 140 in the right and left eyes, respectively). Previous reports confirmed the accordance of peripapillary vessel dropout and RNFL loss in different acute and chronic optic neuropathies including glaucoma and non-arteritic ischemic optic neuropathy (NAION),^[8,14]^ so lower temporal vessel density can indicate higher axonal damage in this important area.

In MTON, formate toxicity inhibits the mitochondrial function through inhibition of the cytochrome oxidase system. Production of reactive oxygen species and toxic aldehydes exacerbate mitochondrial damage.^[15]^ Therefore, MTON can be considered an optic neuropathy with both direct mitochondrial damage of endothelial cells of capillaries and secondary autoregulatory reduction of peripapillary vessels. Another striking finding in our patient was the lower vessel density in the temporal quadrant of the NLP eye; this finding could strengthen the hypothesis of direct mitochondrial damage of the vessels in the event of methanol poisoning. RNFLs of the papillomacular bundle which is directly responsible for central visual acuity contain more vulnerable and smaller fibers. Previous studies confirmed the order of RNFL involvement in Leber hereditary optic neuropathy (LHON), a disease of mitochondrial involvement, first affects the temporal quadrant and lastly the nasal quadrant.^[16]^

In RP, vascular damage is the early event and optic neuropathy can be considered as a secondary event resulting from peripapillary vessel attenuation and photoreceptor degeneration.^[9]^ In our RP patient, optic pallor was obvious, yet the vessel density was higher in all quadrants as compared to the MTON patient. Although the lower RP vessel density compares to the MTON in only one case which might not be conclusive, it could imply that both mechanisms are connected in MTON. In other words, RNFL, GCL, and vascular endothelial cell destruction together accentuate the damage of vessels and these could explain why RPC vessel dropout is more severe in MTON.

OCT-A is a safe and fast method to evaluate retinal vessels in survivors of methanol poisoning in whom FA may be contradicted. Spaide et al comparing FA and OCT-A in 12 patients with different optic neuropathies concluded OCT-A outperforms FA in the visualization of the precapillary network.^[17]^ Moreover, the quantitative measurement of ONH vessels is possible with OCT-A rather than FA. Nevertheless, many patients with NLP cannot be evaluated by OCT-A as a result of poor fixation.

In conclusion, MTON as a rare and devastating optic neuropathy can be further evaluated by OCT-A. Our report demonstrated a reduction in RPC vessel density for the first time. Future studies are needed to confirm our findings in a series of patients.

##  Financial Support and Sponsorship

Nil.

##  Conflicts of Interest

There are no conflicts of interest.
